# High ellipticity reduces semi-circular canal sensitivity in squamates compared to mammals

**DOI:** 10.1038/s41598-019-52828-9

**Published:** 2019-11-11

**Authors:** Jana Goyens

**Affiliations:** Laboratory of Functional Morphology, University of Antwerp, Universiteitsplein 1, 2610 Antwerp, Belgium

**Keywords:** Evolution, Biomechanics, Computational models, Sensory processing

## Abstract

The semi-circular canals in the inner ear sense head rotations. It is widely recognised that the anatomy of the semi-circular canals is often adapted to the species-specific agility, in order to provide the necessary sensitivity. Based on research on mammals, the ellipticity of the semi-circular canal was so far considered as a non-important factor herein. A dataset of 125 squamate species and 156 mammalian species, now shows that the posterior semi-circular canal of squamates is much more elliptical (eccentricities ranging between 0.76 and 0.94) than that of mammals (eccentricities ranging between 0 and 0.71). Fluid-Structure Interaction computer models show that the effect of the ellipticity on sensitivity is strongest in small semi-circular canals. This new insight indicates that the high ellipticity in squamates leads to a severe reduction in sensitivity of up to 45%. In mammals, on the other hand, the reduction in sensitivity is limited to 13%, which is consistent with previous literature that found a limited effect of semi-circular canal ellipticity in mammals. Further, there is a strongly negative correlation between semi-circular canal size and eccentricity in squamates, which is absent in mammals. Hence, the smallest squamates have the most elliptical semi-circular canals. In general, the smaller the semi-circular canal, the less sensitive it is. Therefore, the highly elliptical squamate canals are probably the result of fitting the largest possible canal in small and flat head. Miniaturising the canals while maintaining a circular shape would reduce the sensitivity by another 73% compared to the highly elliptical canals.

## Introduction

The semi-circular canals in the inner ear provide a sense of head rotation to the brain that is indispensable for a stable gaze, balance during locomotion, and a steady frame of reference in the brain onto which the other senses are mapped^1^. The sensitivity of the semi-circular canals depends on their size and shape^2,3^. For example, the size^4–9^, duct diameter^10,11^, and anatomical variation^12,13^ of the semi-circular canals have been found to be adapted to the sensitivity required by the animal’s agility and locomotion style. It is unclear whether also the ellipticity of the semi-circular canals is important. On the one hand, correlations have been found between semicircular canal ellipticity and agility^14–17^. But on the other hand, the results are contradictive^18,19^, only small deviations from circularity have generally been found, and the observed deviations are too small to decrease the sensitivity of the canals substantially^20,21^. A large comparison over a wide range of taxa could provide elucidation, but has not been performed, yet.

**Figure 1:**
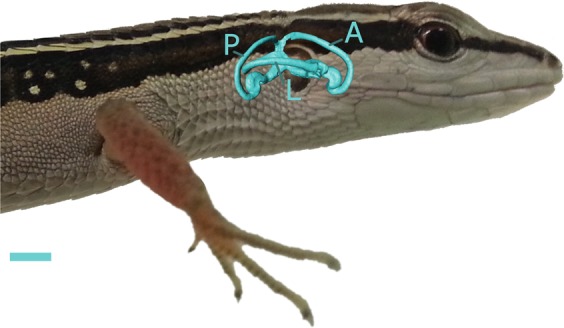
Lateral view on a surface model of the membranous labyrinth of Takydromus sexlineatus (eccentricity: 0.90). The posterior (P), anterior (A) and lateral (L) canal are indicated. The scale bar indicates 1 mm. Photo credit: Charlotte Van Moorleghem. Image created using GIMP 2.8 (http://gimp.org).

In previous anatomical investigations of the semi-circular canals^15,22^, it caught our attention that lacertid lizards seem to have very elliptical canals compared to the more circular canals of other (mammalian) taxa represented in the literature (see Fig. 1). If measurements confirm that lacertid lizards have, indeed, highly elliptical semi-circular canals, this raises several interesting questions. Is the high ellipticity related to the small size of their head? Is this a property of the Lacertidae family, or is this a more general feature of lizards or even squamates? How does this compare to the ellipticity of the semi-circular canals of mammals? And finally, what is the impact of the highly elliptical semi-circular canals on the sensitivity of the system and how does this relate to the agility of the animals? These questions were investigated by calculating the semi-circular canal ellipticity of a large sample of 125 squamate species and 156 mammalian species.

In very small heads, the semi-circular canal anatomy faces a size constraint. Smaller semi-circular canals are less sensitive, which is why there is a general trend of *relatively* larger semi-circular canals in smaller animals (i.e. a general negative allometry^4,23^), in order to maintain sufficient sensitivity. In very small animals, the semi-circular canals fill a substantial part of the skull^24^. It is well possible that more elliptical canals are the result of fitting large canals in this confined space^21^. This reasoning predicts a negative correlation between head size and ellipticity. In a diverse sample of placental mammals, Cox and Jeffery^21^ found, however, no such correlation. This may be due to the fact that the deviations from circularity are small in mammals^21^. Hence, such a negative correlation is hypothesized to exist in squamates if they indeed have highly elliptical canals.

Regarding the impact of the semi-circular canal ellipticity on its sensitivity, it has been stated that small deviations from circularity hardly affect the plane surface area of the canal, and therefore hardly decrease the sensitivity^3,20^. The small reduction in sensitivity would easily be compensated by a wider canal^20^. However, the sensitivity equation by Oman *et al*.^3^ on which McVean^20^ based its conclusions, also shows that the sensitivity does, in fact, reduce strongly when the ellipticity becomes extreme. If lizards are in this situation, which is hypothesized, this would have a substantial negative consequence on the sensitivity of their semi-circular canals. Since lizards are generally fast, maneuverable and agile runners, a reduced sensitivity for head rotation may well pose a large drawback to their locomotion and, indirectly, their fitness. However, the sensitivity equation by Oman *et al*.^3^, which is based on the classic Stainhausen/Groen description of the endolymph flow within the semi-circular canal, does not take into consideration that the effect of the ellipticity may be dependent on the size of the canal. Indeed, the lizard canals are so small that they have a very low Reynolds number (Re_lizard_ ≈ 0.06 < Re_human_ ≈ 0.3; estimations based on computer models, see further), which affects the fluid mechanics of the system. Hence, an in-depth investigation of the influence of ellipticity on sensitivity for a range of semi-circular canal sizes, will be performed using Fluid-Structure Interaction computer simulation models. These models calculate the deformation of the sensory membrane (the cupula) within the posterior semi-circular canal caused by the interaction of the cupula with the endolymph fluid in the canal. Since this deformation defines the stimulation of the hair cells in the cupula, the models give a very precise measure of the sensitivity of the system.

## Material and Methods

### Ellipticity measurements

#### Eccentricity, circularity and radius of curvature

**Figure 2:**
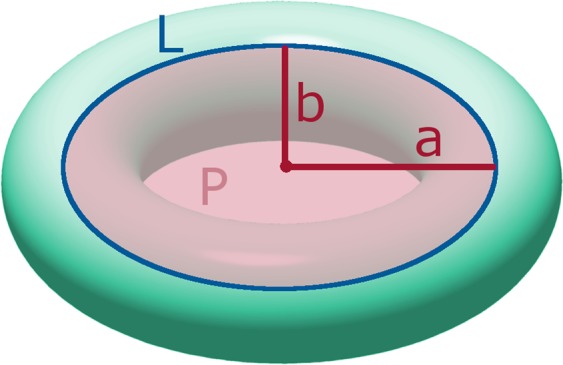
Ellipse with indication of the streamline length (L), the plane area (P), and the semi-major (**a**) and semi-minor (**b**) axes. Image created using GIMP 2.8 (http://gimp.org).

Two variables are in use to describe how elliptical (oval) the semi-circular canals are: circularity and eccentricity^20,21,25,26^. Circularity is defined as:

1 $$circ=(\frac{2P}{L})/R$$

with *L* the streamline length of the fluid flow (i.e. the length of the canal), *P* the plane area enclosed by *L*, and *R* the radius of curvature of the canal (see Fig. 2). The radius of curvature R is defined as the average of the semi-major (a) and the semi-minor (b) axes the canal (see Fig. 2). The circularity has a value of 1 for a perfect circle, and a value smaller than 1 for ellipses^21^. Eccentricity is defined as:

2 $$ecc=\sqrt{1-\frac{{b}^{2}}{{a}^{2}}}$$

The eccentricity is 0 for a perfect circle (when a equals b), and has a value between 0 and 1 for ellipses. The higher the value, the flatter the ellipse. We mainly use the eccentricity in this investigation, as the information that is necessary for its calculation is more often available in the literature.

#### Calculation for different taxa

Lacertid lizards. The semi-axes a and b of the semi-circular canals, necessary to calculate the eccentricity, were determined using the landmarks of Vasilopoulou-Kampitsi *et al*.^15^ for 23 lacertidae species (one specimen per species). These landmarks were obtained in ISE-Mesh Tools 1.3.3 (http://morphomuseum.com/tutorialsMeshtools)^27^, based on microCT scans of the bony labyrinth. Five homologous landmarks and 28 semi-landmarks describe the centreline curve of the posterior semi-circular canal and the common duct. Based on these landmarks, the semi-axes a and b were determined in Matlab (Matlab R2018b, MathWorks, Natick, MA, USA). The direction of the semi-minor and semi-major axes in the plane of the canal were calculated using a local frame of reference. Next, a spline was fitted through the landmarks, and the length of the semi-minor and semi-major axes (a and b) of this spline were calculated. The streamline length L was defined as the sum of the Euclidian distances between the landmarks, and the plane area P as the area of the polygon specified by the landmarks in the plane of the canal. Finally, the radius of curvature R, the eccentricity and the circularity were determined.

Anole lizards. The procedure described above was used to calculate the radius of curvature R, the eccentricity, and the circularity for 41 *Anolis* species, based on landmark data that Dickson *et al*.^28^ provided in their supplementary material. Dickson *et al*.^28^ segmented microCT scans in Materialise Mimics software, and subsequently derived 34 landmarks describing the centreline curves of the posterior semi-circular canal and the common duct, in the ‘medcad’ module in Mimics.

Snakes. The radius of curvature R, the eccentricity, and the circularity were calculated in the same way for 61 snake species, based on the landmark data provided by Palci *et al*.^29^ in their supplementary material. Palci *et al*.^29^ segmented microCT scans in Avizo software (version 9.0, Visualization Sciences Group). They described the posterior semi-circular canal and the common duct by a total of 16 landmarks in Landmark Editor (version 3.6; http://www.idav.ucdavis.edu/research/EvoMorph).

Mammals. No landmark data of other taxa were found in the literature, but datasets containing semi-axes are available. The length and width of the posterior semi-circular canals of 17 xenarthran species (40 specimens in total) were measured by Billet *et al*.^30^, 36 placental mammal species by Ekdale^16^, 49 species of the squirrel-related clade by Pfaff *et al*.^10^, 3 ruminant species (25 specimens in total) by Mennecart and Costeur^31^, 33 marsupial species by Pfaff *et al*.^11^, and 17 carnivore species (20 specimen in total) by Schwab *et al*.^32^. The 8 species that were in included by Pfaff *et al*.^10^ for comparative purposes were included in the placental mammals group. The semi-major and semi-minor axes a and b were obtained by dividing the semi-circular canal length and width by two, and were used to calculate the radius of curvature R and the eccentricity of the semi-circular canals.

#### Statistical analyses

A statistical analysis was used to test whether the slope between the radius of curvature R and the eccentricity was less than one, i.e. whether smaller vestibular systems have more eccentric semi-circular canals.

For the lacertid and *Anolis* datasets, the phylogenetic signal of the eccentricity could be assessed. The Bayesian trees of Baeckens *et al*.^33^ and Gamble *et al*.^34^ were pruned for respectively the lacertid and the *Anolis* lizards included in this study, and tested for a phylogenetic signal using the ‘phylosig’ function^35^ of the ‘phytools’ package in R (version 3.5.3,^36,37^). The closer the value of Pagel’s lambda is to zero, the weaker the phylogenetic signal^38,39^. A very weak phylogenetic signal was observed for the eccentricity for both groups (see Table 1), indicating that the eccentricity is not conserved within the phylogeny^38,39^ and that phylogenetic non-independence of the data points does not have to be taken into account.

**Table 1:** Result of the tests for the phylogenetic signal of the eccentricity.

	Lacertidae	*Anolis*
Pagel’s lambda	6.6·10^−5^	6.6·10^−5^
Log likelihood	58	109
p-value	1	1

Linear models were constructed in R to test the correlation between the eccentricity (dependent variable) on the one hand, and the radius of curvature R (fixed factor) and taxon (fixed factor, grouped according to the taxa of the papers from which the data were obtained) on the other hand. Also the interaction between taxon and radius of curvature was included. The significance of the effect of the fixed factors and their interaction was tested using an analysis of variance (ANOVA). A drawback of using literature datasets is that some groups are more closely related than others. Yet, this is mitigated by the very weak phylogenetic dependency of the eccentricity, and because this approach enables a large sample size.

### Mechanical models

#### Sensitivity equation

Oman *et al*.^3^ derived a formula for the mechanical sensitivity of semi-circular canals, from a second-order differential equation that they constructed based on the classic Stainhausen/Groen description of the endolymph flow in a semi-circular canal:

3 $${sensitivity}=\frac{P}{4\pi \cdot L\cdot {A}_{c}\cdot \nu \cdot \frac{\bar{S}}{A^{2}}}$$

with $\overline{S/A^{2}}$ the average of the ratio of the local flow drag wall shape factor S (calculated using the measurements by Curthoys *et al*.^40^) to the squared cross-sectional area A of the semi-circular canal; A_c_ the cross-sectional area of the cupula; and ν the kinematic viscosity of the endolymph (1·10^−6^ m²/s^41^).

Equation 3 was used to estimate the sensitivity of canals with eccentricities of 0, 0.47, 0.71, 0.86 and 0.94 (see Fig. 5 and Supplementary Fig. 1). This comparison was made for canals of four different sizes: a lizard-sized canal (R = 0.61 mm, see further), a canal with a size intermediate between lizard and human (R = 1.9 mm, the size of e.g. domestic cats), a human-sized canal (R = 3.2 mm^40^), and a canal larger than human (R = 4.4 mm).

#### Fluid-Structure Interaction models

The influence of canal size and eccentricity on the sensitivity of the semi-circular canals was also determined more precisely using Fluid-Structure Interaction models. For a detailed description of the model, its convergence analysis, sensitivity analysis, and validation, please refer to Goyens *et al*.^42^ and Goyens & Aerts^43^. Below, a short overview of the model is given, as well as the properties that are specific to this investigation. This investigation takes a reductionist approach, in which all parameters are kept constant, except for the radius of curvature and the eccentricity.

Geometry. The geometry was a simplified version of the membranous labyrinth anatomy measured by Curthoys *et al*.^40^ in humans. The geometry consisted of a semi-circular canal with a 3.2·10^−4^ m wide narrow duct, and a wider chamber (the ampulla). A 4.03·10^−4^ m thick cupula was located in the centre of the ampulla (see Supplementary Fig. 1). Five versions of this geometry were constructed, with eccentricities of 0, 0.47, 0.71, 0.86 and 0.94. Subsequently, these human-sized geometries were scaled to the sizes listed in the Sensitivity equation section, arriving at a total of 20 geometries. Finally, an additional circular geometry was constructed with a radius of curvature equal to the semi-minor axis of the lizard-sized canal (R = 0.31 mm).

Endolymph model. The endolymph fluid in the semi-circular canal was modelled with Computational Fluid Dynamics (CFD) in Ansys Fluent (version 19.1, Pittsburgh, USA). Consistent with the literature, the fluid properties of water were used for the endolymph^41^. Little is known on the endolymph properties, but if it is more viscous in the cold blooded squamates, this may in principle reduce their sensitivity compared to warm-blooded mammals (although this effect is probably small^43^). The walls were modelled as “no slip” walls, which made a rotating movement of 30° in 0.222 s, with a top velocity of 250°/s. This corresponds to the natural human head movements during active everyday activities, such as sprinting, jumping forward, and running in the woods^44,45^.

Cupula model. The cupula deformation during the head manoeuver was modelled with Finite Element Modelling (FEM) in Ansys Transient Structural (version 19.1). Consistent with the literature, a Young’s modulus of 54 Pa and a Poisson ratio of 0.3 were used. The surfaces of the cupula that attach to the membranous labyrinth walls were modelled with a fixed boundary condition, rotating along with the head manoeuver. At the outer edge of the canal, sensory hair cells are embedded in the cupula. These hair cells deflect when the cupula deforms, hence, the calculated cupula strain at this location is a precise measure for the sensitivity at this location.

Endolymph – cupula interaction. At the contact surface between the endolymph and the cupula, forces are exerted by the endolymph on the cupula (output of CFD becomes input for FEM), and the deformation of the cupula induces fluid flow (output of FEM becomes input for CFD). This two-way interaction was modelled in the System Coupling module of Ansys Workbench (version 19.1).

## Results and Discussion

### Extreme ellipticity in lacertid lizards

**Figure 3:**
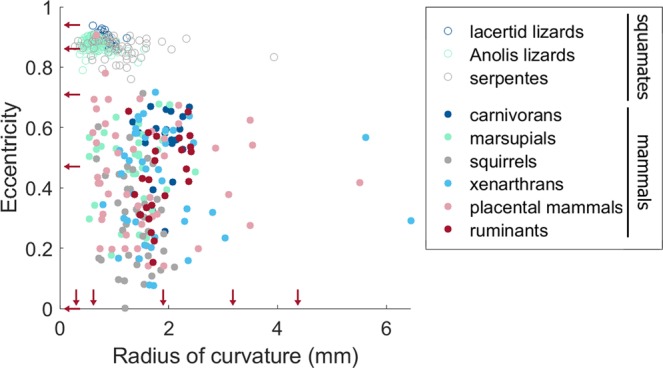
Semi-circular canal eccentricity in function of the radius of curvature (R) in squamates (open symbols) and in mammals (filled symbols). Red arrows indicate the sizes and eccentricities of the computer models. Image created using Matlab R2018b (https://nl.mathworks.com) and GIMP 2.8 (http://gimp.org).

The current measurements confirm the initial impression that lacertid lizards have highly elliptical semi-circular canals. The eccentricity of their canals varies between 0.86 and 0.94 (see Table 2); which are extreme values given that the theoretical maximal value of the eccentricity of an ellipse is 1. The eccentricity of the lacertid lizard canals hardly overlaps with the dataset of the mammals (see Fig. 3). The only mammalian species that we found in the literature with an eccentricity close to, or within, the range of the lacertid lizards, are *Tursiops truncates* (the botllenose dolphin, eccentricity = 0.78) and *Spalax microphtalmus* (the greater mole-rat, eccentricity = 0.91). Perhaps these species are exceptionally because of their aquatic and subterranean lifestyles, however, the few other species in our dataset that exhibit these lifestyles do not have highly elliptical semi-circular canals. Interestingly, though, it has been mentioned in the literature that marine carnivorans tend to have more elliptical semicircular canals than terrestrial ones^16,17,46^, although the exemplary canals depicted by Ekdale^46^ are clearly less elliptical than those observed for squamates and *T. truncates*. Anyhow, if an increased ellipticity is indeed related to an aquatic habitat, the associated reduction in sensitivity is consistent with the observed reduction in semi-circular canal size in cetaceans, which was interpreted as an adaptation for agile swimming in an environment that lacks the restrictions of terrestrial contact^7^. Besides the two discussed outliers, all other mammals that are represented in our investigation, have eccentricities of 0.71 or (much) lower (see Table 2 and Fig. 3).

**Table 2:** Eccentricity, circularity and radius of curvature (R) of squamates and mammals.

	Eccentricity			Circularity			Radius of curvature		
	Min	Max	Av ± sd	Min	Max	Av ± sd	Min	max	Av ± sd
Lacertid lizards	0.86	0.94	0.90 ± 0.02	0.70	0.82	0.76 ± 0.03	0.61	1.24	0.84 ± 0.14
*Anolis* lizards	0.79	0.91	0.88 ± 0.02	0.67	0.90	0.83 ± 0.03	0.36	1.55	0.66 ± 0.19
Snakes	0.76	0.91	0.87 ± 0.03	0.59	0.80	0.71 ± 0.04	0.28	3.93	1.07 ± 0.55
Carnivorans	0.25	0.67	0.55 ± 0.11				1.3	2.4	1.92 ± 0.28
Marsupials	0.18	0.68	0.47 ± 0.13				0.54	2.49	1.33 ± 0.49
Squirrel-related	0	0.71	0.30 ± 0.16				0.67	2.1	1.38 ± 0.35
Xenarthrans	0.076	0.718	0.41 ± 0.18				0.79	6.4	1.9 ± 1.1
Placental mammals	0.14	0.91	0.45 ± 0.19				0.60	5.5	1.6 ± 1.0
Ruminants	0.15	0.65	0.44 ± 0.14				1.3	2.4	1.85 ± 0.37

### Comparison of squamates with mammals

Comparison with *Anolis* lizards and snakes shows that extremely elliptical semi-circular canals are not unique for lacertid lizards. Rather, this appears to be a general characteristic of squamates, all having eccentricities above 0.76 and taxon averages close to 0.9 (see Table 2 and Fig. 3). It was hypothesised that this may be due to space constraints in their small skulls, because a more elliptical shape may enable fitting the semi-circular canal in the very limited available space. But interestingly, this seems not necessarily to be the case. Although a part of the *Anolis* lizards and snakes has smaller canals than any of the included mammals, the highest eccentricities are found for lacertid lizards, whose radius of curvature falls within the size range of mammals (more specifically, the range of the marsupials and the squirrel-related clade; see Table 2 and Fig. 3). Hence, there are mammals with equally small semi-circular canals that nevertheless retained a circular shape. The posterior semi-circular canal is oriented dorso-ventrally, hence a flattened head may “squeeze” the canal in an elliptical shape. It is very well possible that the mammals at the lower end of the size range have less flat heads than the lizards, making the size constraint less severe, but this is a hypothesis that cannot be tested directly with the available data.

### Effect of canal size and head shape on canal ellipticity

**Figure 4:**
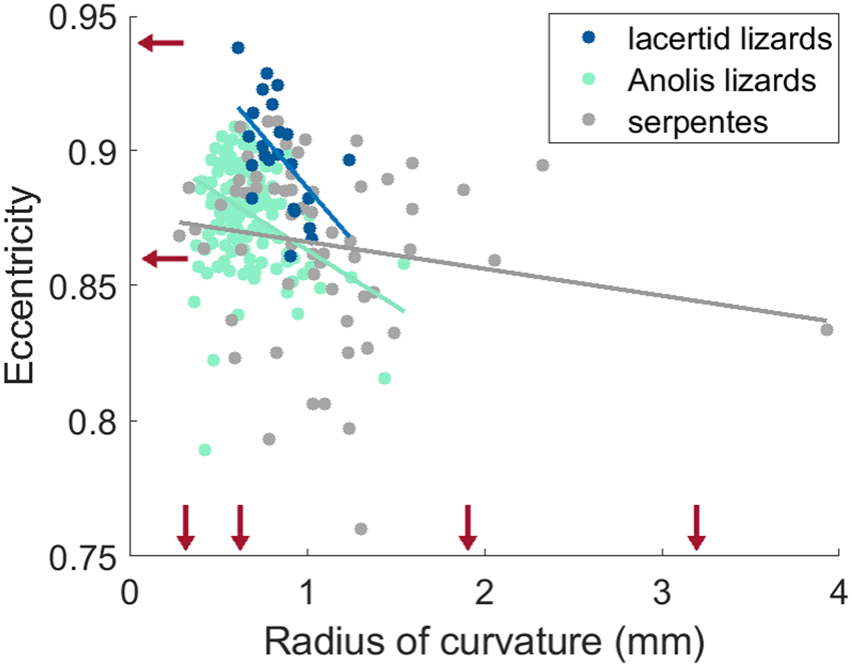
Semi-circular canal eccentricity in function of the radius of curvature (R) in squamates. Red arrows indicate the sizes and eccentricities of the computer models that fall within the range of the squamates. Image created using Matlab R2018b (https://nl.mathworks.com) and GIMP 2.8 (http://gimp.org).

What can be tested, is how the size of a canal affects its ellipticity within a taxon; i.e. whether species with smaller semi-circular canals have higher eccentricities than other species in their taxon. Consistent with the literature^21^, such a relationship is absent in all mammalian taxa: the slope is not different from zero (slope: −0.00089 ± 0.02508 SE; p-value: 0.66), and this slope is not different between taxa (p-value: 0.51). The opposite is true for squamates: here, the negative slope is strongly significant (slope: −0.041 ± 0.017 SE; p-value: 0.0003), and again this slope is not different between taxa (p-value: 0.12). Hence, smaller squamate canals are indeed more elliptical (see Fig. 4). The size constraint in their heads is so severe that the canals become dorso-ventrally compressed, possibly because their heads are more dorso-ventrally flattened than those of mammals^47^. A flat head may enable squamates to hide from predators in small crevices^48^, but it may at the same time reduce bite force capacity in lizards^49^ and (see further) the semi-circular canal sensitivity.

### Effect of ellipticity on sensitivity in squamates

**Figure 5:**
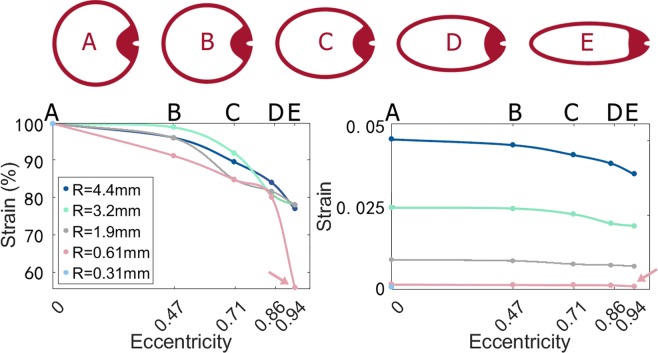
Effect of semi-circular canal eccentricity and size on the strain in the cupula for models with five different sizes (different radii of curvature, R). Strain is a dimensionless measure for sensitivity. Left: strain values relative to the strain in the circular canal. Right: absolute strain values. Arrows indicate the lizard-sized model with the highest observed eccentricity. Letters correspond to the five different eccentricities that are modelled, whose model outlines are shown in red above the graphs. The red outlines are depicted at the same radius of curvature. Image created using Microsoft Excel 2016 (https://products.office.com/en/excel) and GIMP 2.8 (http://gimp.org).

The final question that remains, is what influence this dorso-ventral compression has on the sensitivity of the semi-circular canals. Many of the included squamate species are active and agile (all of the lacertid species are), excluding the possibility that they simply do not need sensitive semi-circular canals and the benefits this gives for gaze stabilisation, balance during locomotion, and a stable reference frame for the brains^1,4,24^. Also, if there was no need for functional semi-circular canals, squamates could just as well have had highly circular miniature canals, rather than very elliptical canals that presumably take as much space in the skull as possible. The sensitivity equation by Oman *et al*. (Eq. 3)^3^ predicts that the sensitivity of a semi-circular canal strongly reduces with increasing eccentricity, but only for extreme eccentricities. This is the case in squamates: for an eccentricity of 0.94, which is the highest eccentricity found, the sensitivity is decreased by 32% compared to a circular-shaped canal with the same radius of curvature, according to Eq. 3. However, this calculation does not yet take into account the potential effect of semi-circular canal size. The computer simulation models (see Supplementary Fig. 1) show that the effect of the semi-circular canal eccentricity on the sensitivity indeed depends on radius of curvature. This effect is non-linear: especially in very small semi-circular canals, the sensitivity abruptly declines at the highest eccentricities (see Fig. 5). As a result of this new insight, an eccentricity of 0.94 is estimated to decrease the sensitivity by 45% compared to an equally small, but circular, semi-circular canal (i.e. with the same radius of curvature). Hence, the current results contradict the literature that states that semi-circular canal ellipticity hardly influences sensitivity^3,20,21^, because (1) squamates have much higher eccentricities than mammals, and (2) the effect of the eccentricity on sensitivity is amplified due to the small size of their semi-circular canals. The reduction in sensitivity is severe, showing that adopting a highly elliptical shape in order to fit the semi-circular canals in a small, flat head, comes at a cost in terms of sensitivity for the squamates. Yet, this elliptical shape is still much less costly than resizing the semi-circular canal while maintaining a circular shape. A circular canal with a radius of curvature equal to the semi-minor axis of the most eccentric squamate canal (R = 0.31 mm, i.e. the largest circular canal that fits within the most eccentric squamate canal), would only have 73% of the sensitivity of the latter.

### Effect of ellipticity on sensitivity in mammals

Mammals have much lower semi-circular canal eccentricities than squamates, usually ranging between 0 and 0.71 (see Fig. 3). Both the sensitivity equation by Oman *et al*. (Eq. 3^3^); and the computer models (see Fig. 5) predict that this will only lead to relatively small decreases in sensitivity (a maximal decrease of respectively 4.6% and 13%), because the decrease in sensitivity remains minor for small to moderate deviations from circularity. Hence, the current anatomical measurements and sensitivity calculations confirm previous findings in the literature^21^ that, *in mammals*, the deviation from circularity is usually much too small to have a severe impact on the sensitivity of the system.

## Conclusions

The ellipticity of the semi-circular canals was compared between squamates and mammals. A large dataset shows that squamate canals are much more elliptical than those of mammals. With the exception of one aquatic and one subterranean mammal, the range of eccentricity does not overlap between both groups. Only in squamates, a negative correlation is found between canal size and canal ellipticity. This may well be related to a more flattened head shape in squamates^47,48^, enabling hiding from predators in small crevices. Computer models show that the canal ellipticity decreases the canal sensitivity the strongest in small, squamate-sized canals. As a result, the sensitivity of the highly elliptical squamate semi-circular canals is reduced by 45% compared to a circular-shaped canal of the same size. However, adopting an elliptical size to “squeeze” the canal in a small, flattened head is still beneficial compared to miniaturising the canal while keeping a circular shape, which would reduce the sensitivity by another 73% compared to the squamate-sized elliptical canal.

## Supplementary information

Supplementary information

Supplementary information

## Data Availability

The data used in this investigation are available in the supplementary material.
