# Supplementary material for: High ellipticity reduces semi-circular canal sensitivity in squamates compared to mammals
Source: Sci Rep. 2019 Nov 11;9:16428. doi: 10.1038/s41598-019-52828-9 (PMC6848070; doi:10.1038/s41598-019-52828-9)
Supplement: Supplementary file 1 — Supplementary information [file 41598_2019_52828_MOESM1_ESM.pdf]

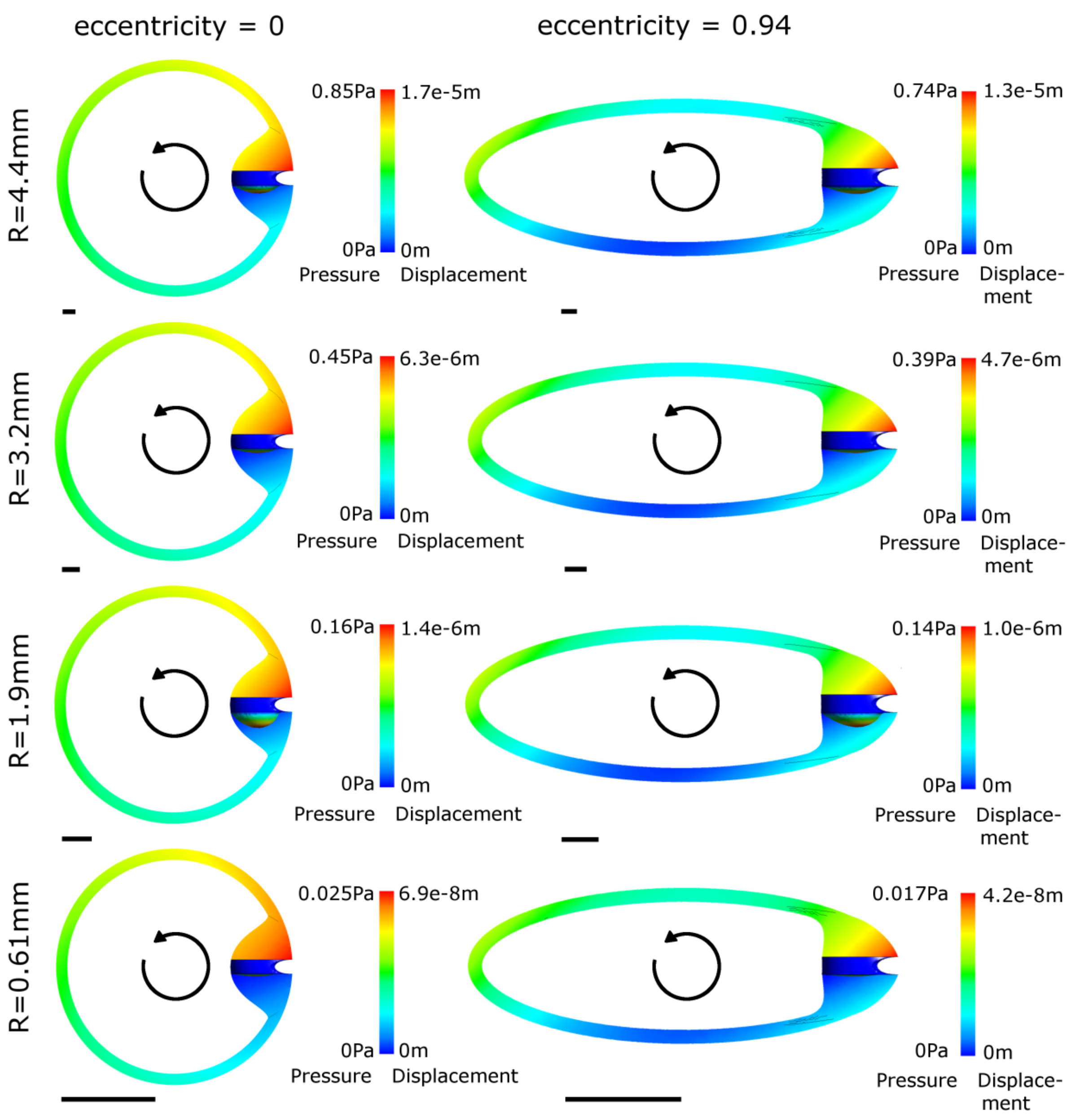

Supplementary figure 1: Outcomes of the Fluid-Structure Interaction computer models. Endolymph fluid pressure and the deformation of the cupula are given at t=0.1s for models of 4 different sizes, both for the circular shape (eccentricity=0) and the most elliptical shape (eccentricity=0.94). Scale bars indicate 0.5mm. The arrows show the direction of the head manoeuver. The cupula deformation is enlarged 20 times for the two largest models, and 200 times for the two smallest models for visualisation purposes.
